# Simulation of a control method for active kinesiotherapy with an upper extremity rehabilitation exoskeleton without force sensor

**DOI:** 10.1186/s12984-024-01316-x

**Published:** 2024-02-11

**Authors:** Piotr Falkowski, Kajetan Jeznach

**Affiliations:** 1 ŁUKASIEWICZ Research Network - Industrial Research Institute for Automation and Measurements PIAP, Al. Jerozolimskie 202, 02-486 Warsaw, Poland; 2 Warsaw University of Technology, Pl. Politechniki 1, 00-661 Warsaw, Poland

**Keywords:** Exoskeleton, Kinesiotherapy, Fuzzy controller, Rehabilitation robotics, Sensorless control, Movement support, 0000, 1111

## Abstract

Exoskeleton-aided active rehabilitation is a process that requires sensing and acting upon the motion intentions of the user. Typically, force sensors are used for this. However, they increase the weight and cost of these wearable devices. This paper presents the methodology for detecting users’ intentions only with encoders integrated with the drives. It is unique compared to other algorithms, as enables active kinesiotherapy while adding no sensory systems. The method is based on comparing the measured motion with the one computed with the idealised model of the multibody system. The investigation assesses the method’s performance and its robustness to model and measurement inaccuracies, as well as patients’ unintended motions. Moreover, the PID parameters are selected to provide the optimal regulation based on the dynamics requirements. The research proves the presented concept of the control approach. For all the tests with the final settings, the system reacts to a change in the user’s intention below one second and minimises the changes in proportion between the system’s acceleration and the generated user’s joint torque. The results are comparable to those obtained by EMG-based systems and significantly better than low-cost force sensors.

## Introduction

Physiotherapy is the process of recovering the maximum available physical performance of a patient with a certain level of impairment [1, 2]. It can be realised as active or passive treatment [3]. While patients with lighter motion disorders can even recall their motion capabilities and connect them with genuine neurological patterns, those with severe disabilities require mobilising their joints to prevent them from stiffness [1].

The treatment can be partially automatised and supported with robotics technology. Today, there are many commercially available physiotherapeutic devices [3]. However, new technologies of rehabilitation are being developed and introduced into clinical practice [4].

In general, rehabilitation robots are used in physiotherapy to reduce therapists’ physical effort during complex exercises [5], increase the frequency and length of the sessions [6] and improve the accuracy of repeatable exercises [7]. Moreover, they enable precise performance measurements and, hence, constant feedback during therapy [8].

Rehabilitation robots are typically either end-effector open-chain structures or exoskeletons. In upper-extremity rehabilitation, the former are usually used to lead the patient’s hand [9], while the latter act in parallel to the extremity [10]. The first ones do not allow for the direct mobilisation of single joints [9]. Therefore, the therapy with them does not control whether the performed motion follows the corresponding anatomical pattern. On the contrary, exoskeletons are vulnerable to dimensional differences and misalignments relative to the user’s extremities. These caused when designing or installing the device can cause injuries or inaccurate exercising [10].

Robot-aided kinesiotherapy involves support or resistance of the rehabilitation device [2]. However, for this, the motion intention of a patient must be detected. The detection can be realised with either mechanical signals, including direct force measurements, or other techniques correlating biosignals; electromyography (EMG), among others [11, 12].

The EMG-based methods can either be correlated with the muscular groups responsible for the intended movements [13, 14] or with other groups [15]. Among others, the latter gives the opportunity to control the devices supporting impaired extremities by the other hand’s gestures [15]. On the contrary, some of the described methods aim at recalculating registered EMG into joint torques [16].

The mechanical signals-based approach can use tactile or force sensing performed with different components and different analysis algorithms [17]. Nevertheless, it is typically used with the external force sensors; as for the hand guidance of cobots [18, 19]. Typically, implementing commercial force sensors into the design increases the cost and weight of the devices equipped with these [20]. On the other hand, EMG tracking is severely vulnerable to individual anatomic differences and electric noises [19].

The study aims to develop and validate the methodology of detecting and reacting to users’ intentions without force sensors nor biosignals tracking devices [21–23]. Within the presented investigation, only the 14-bit encoders integrated with the servodrives are used [24]. Therefore, thanks to such an approach, a lightweight and low-cost exoskeleton can be used for active physiotherapy involving a user in the feedback loop [3]. This is targeted at increasing the availability of exoskeleton-based systems and their safety. Hence, to achieve the minimally-supervised therapy [25, 26]. Moreover, it can be used for lightweight assistive human exoskeletons [27, 28].

Currently, no low-cost and lightweight technologies with a short time of detecting users’ intentions are developed. This means that the presented approach is unique in using only the sensors already integrated with the drives and hard-coded algorithms. The investigation results will be implemented practically in the ExoReha exoskeleton developed by ŁUKASIEWICZ Research Network - Industrial Research Institute for Automation and Measurements PIAP [29–31].

## Methodology

The developed method is based on comparing the simulated motion of the controlled exoskeleton with the real-life values measured with encoders. For this reason, the dynamics computation for a multi-body system must be performed. The differences between the expected motion and the actual ones will be interpreted as a volunteer activity of the user. While detecting such, the drives corresponding to the motion have to react and act based on the identified motion intention. However, the described differences can also result from the unintended activity of an impaired user with coordination problems [32], model inaccuracies or other noises in the control system. The investigation aims to analyse the impact of the method’s parameters and assess its vulnerability to the presented disruptions. Therefore, a set of simulations were run to select the best-performing, robust settings. The simulation approach was selected as safe to use for developing innovative exoskeletons’ control methods based on multibody extremity models [33].

All the works were conducted as a simulation in *Matlab R2021a / Simulink* software. These included computations of the modelled multibody idealised system but also a simulation of the real-life system. The latter is used instead of measuring the real-life system with physical sensors, i.e. encoders in the joints. The second model is designed similarly to the idealised one but also includes external non-measured forces and non-measurable inaccuracies between the model and the real-life system. The motors were designed as *Simulink* blocks with an input for the set torques and an output for the rotor’s angular velocity. The real-life model also had an input for external torque exaggerated by a patient.

The investigation presented in this paper was performed for a single-joint control for the ExoReha exoskeleton’s elbow flexion/extension. The device itself has three active degrees of freedom and two passive. Therefore, a simple multibody model was constructed [30]. It consisted of a motor, two rigid exoskeleton parts and attached to a forearm with a hand, without a possibility of wrist motion.

Two models of motors were built - one reflecting the real-life operation and one idealised. These both were based on the same principle, that the engine torque $M_{motor}$ balances the torques acting against it [34]. These are the $M_a$ torque associated with the angular acceleration of the engine, $M_B$ torque associated with damping and the load torque $M_l$. The Eq. 1 represents the above assumption, while the Eq. 2 extends it with a physical interpretation, where:

- $K_t$ – mechanical constant of a motor;
- $i_w$ – current in the motor’s winding [A],
- *J* – reduced moment of inertia of the driven multibody system [kg$\cdot$m^2^],
- $\omega _s$ – rotor angular velocity [rad/s],
- *B* – damping factor [Nm$\cdot$s/rad],
- $M_{l}$ – engine load torque [Nm]; i.e. reaction of the exoskeleton’s user. While both the loading moment and the rotor’s generated torque are positive or negative, the device is supporting the patient’s intended motion.

1 $$\begin{aligned} M_{motor}= & {} M_a + M_B + M_l, \end{aligned}$$

2 $$\begin{aligned} K_t i_w= & {} J \frac{\text {d}\omega _{s}}{\text {d}t} + B \omega _s + M_{l}, \end{aligned}$$

Transformation of Eq. 2 results in formula 3, which describes the angular acceleration of the rotor.

3 $$\begin{aligned} \frac{\text {d}\omega _{s}}{\text {d}t} = \frac{K_t i_w}{J} -\frac{B \omega _s}{J} - \frac{M_l}{J}. \end{aligned}$$

It is assumed that the initial velocity of the exoskeleton joints controlled by the algorithm is equal to zero. Therefore, Eq. 3, after Laplace transform, is in the form of the formula 4. This can be further simplified to formula 5, which describes the angular velocity of the rotor. Such a form of equation was used in further simulations of the drive’s dynamics and implemented to *Simulink* models.

4 $$\begin{aligned}{} & {} s\omega _s(s) - \underbrace{\omega _s(0)}_{\textrm{0}} = \frac{K_t i_w(s)}{J} -\frac{B \omega _s(s)}{J} - \frac{M_l(s)}{J}, \end{aligned}$$

5 $$\begin{aligned}{} & {} \omega _s(s) = \frac{K_t i_w(s) - M_l(s)}{Js + B}. \end{aligned}$$

**Fig. 1:**
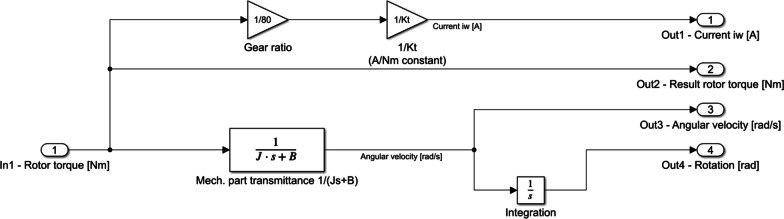
Ideal motor model implementation in *Simulink*

**Fig. 2:**
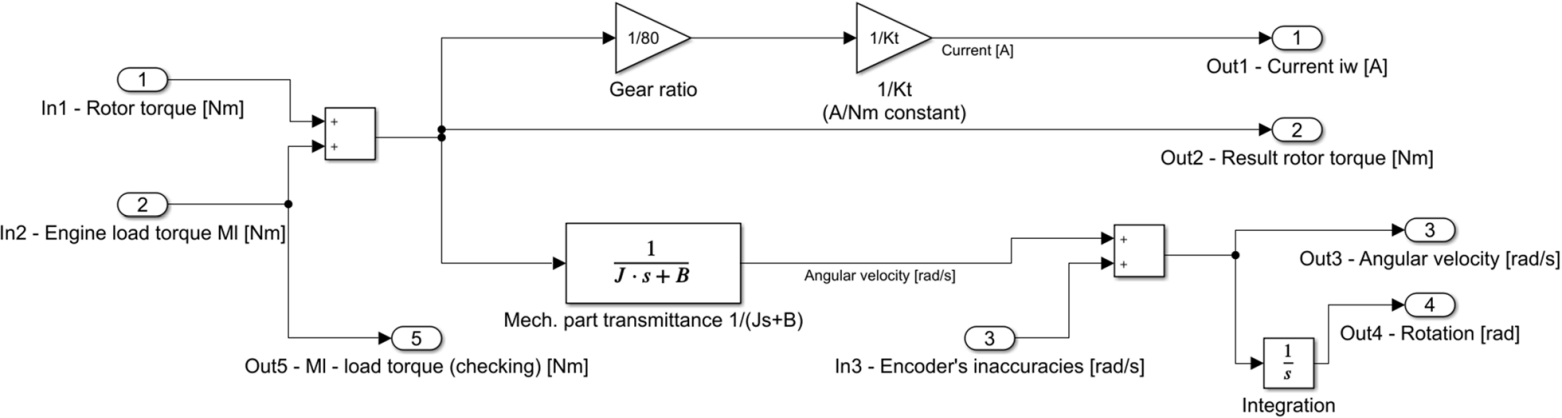
Real motor model implementation in *Simulink*

Implementations of these two models in *Simulink* are presented in Figs. 1 and 2.

For the experimental validation of the system’s performance, data from the *T-MOTOR AK80-9* documentation, mobilising elbow joint in *ExoReha*, were used. These are $K_t = 0.095\ \frac{Nm}{A}$ and $B=0.6\ \frac{Nm}{rad}$. However, $K_t$ is not directly involved in torque control over the device. The moment of inertia acting upon the motor axis was estimated with Stainer’s theorem based on the data from CAD models and anthropometric data for the 95^th^ percentile of male adult population [30, 35] confirmed by literature examples [36]. Its value was taken as $J=0.35\ kg\cdot m^2$.

**Fig. 3:**
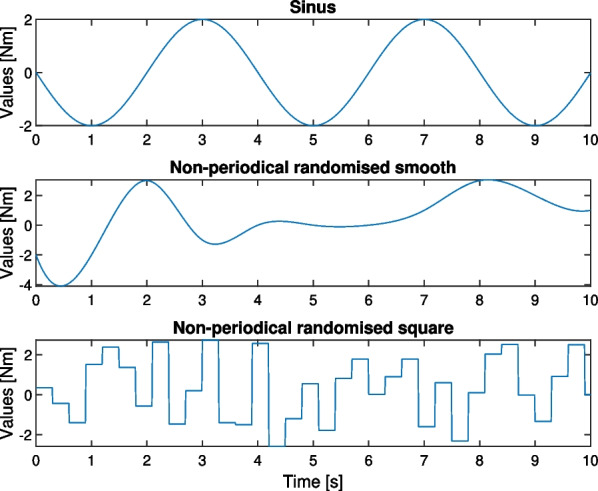
Input signals representing intended torques of the user in their elbow joint

During the investigation, the performance of the control systems was compared for three different input types (sinus, non-periodical randomised smooth and non-periodical randomised square), representing the patient’s torque in an elbow joint (see Fig. 3). The randomised functions were generated with *spline* and *rand* *Matlab* functions correspondingly. Their amplitude maxima were assumed as $4\ Nm$ while the differences between the intended torque and the one obtained by a patient were taken as $0.01\ Nm$ for a standard level and $0.1\ Nm$ for a high level. This parameter can also be used to include potential differences between intended and achieved torques resulting from other factors such as friction or additional low external forces. The input signals were generated with the frequency of $100\ Hz$. Moreover, the test cases included inaccuracies in the modelled *J*, *B*, and $K_t$ parameters, as well as differences between the motion intention of a user and the exaggerated torque and the encoder’s inaccuracies. All the test cases are presented in Table 1.

**Table 1:** Test cases for simulations ($J_m$ - value of the modelled *J* parameter, $J_r$ - value of the real-life *J* parameter, $B_m$ - value of the modelled *B* parameter, $B_r$ - value of the real-life *B* parameter)

Test case	*J* model inaccurcy	*B* model inaccuracy	$M_l$ noise [Nm]	Encoder inaccuracy [rad/s]
1	$J_m=J_r$	$B_m=B_r$	0.01	0.001
2	$J_m=3J_r$	$B_m=B_r$	0	0
3	$J_m=0.5J_r$	$B_m=B_r$	0	0
4	$J_m=J_r$	$B_m=B_r+0.3$	0	0
5	$J_m=J_r$	$B_m=B_r-0.1$	0	0
6	$J_m=J_r$	$B_m=B_r$	0.1	0.001
7	$J_m=J_r$	$B_m=B_r$	0.01	0.01

**Fig. 4:**
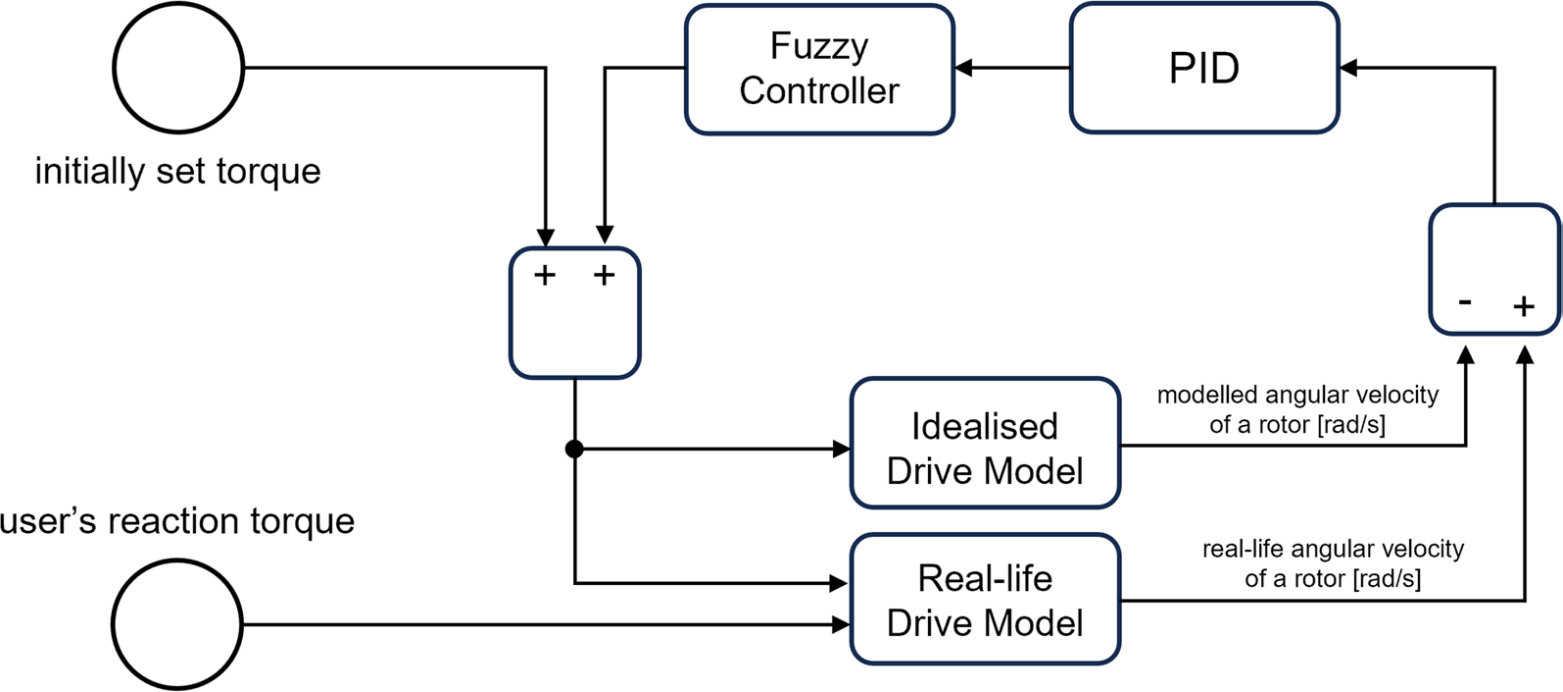
Control algorithm schematic implementation in *Simulink*

The aim of the designed control system is to detect the motion intention of a user and support it with the motors proportionally to the torque generated in the extremity’s joints. However, no direct torque sensation is used. It is assumed that at the beginning of the experiment, no motion is realised, and a patient can exaggerate no muscular force. Therefore, the test sequence with the motor torque of $2\ Nm$ is generated for the first $0.25\ s$. Then, the idealised model is computed as there is no patient’s external force, and the real-life model is simulated. The differences between the computed angular velocities of the rotors are sent to the PID and following fuzzy controllers. The computed control signal is limited if it exceeds the ranges available for the motors and is set as the intended torque at the next timestamp. Schematic implementation of these in *Simulink* is presented in Fig. 4. The accuracies of measuring positions of the rotors are taken as $\pm 0.001\ rad/s$ for a standard level and $\pm 0.01\ rad/s$ for a high level. Non-periodical external torques of patients and all non-stationary inaccuracies in the models are generated randomly to simulate the real-life operation of the system.

**Fig. 5:**
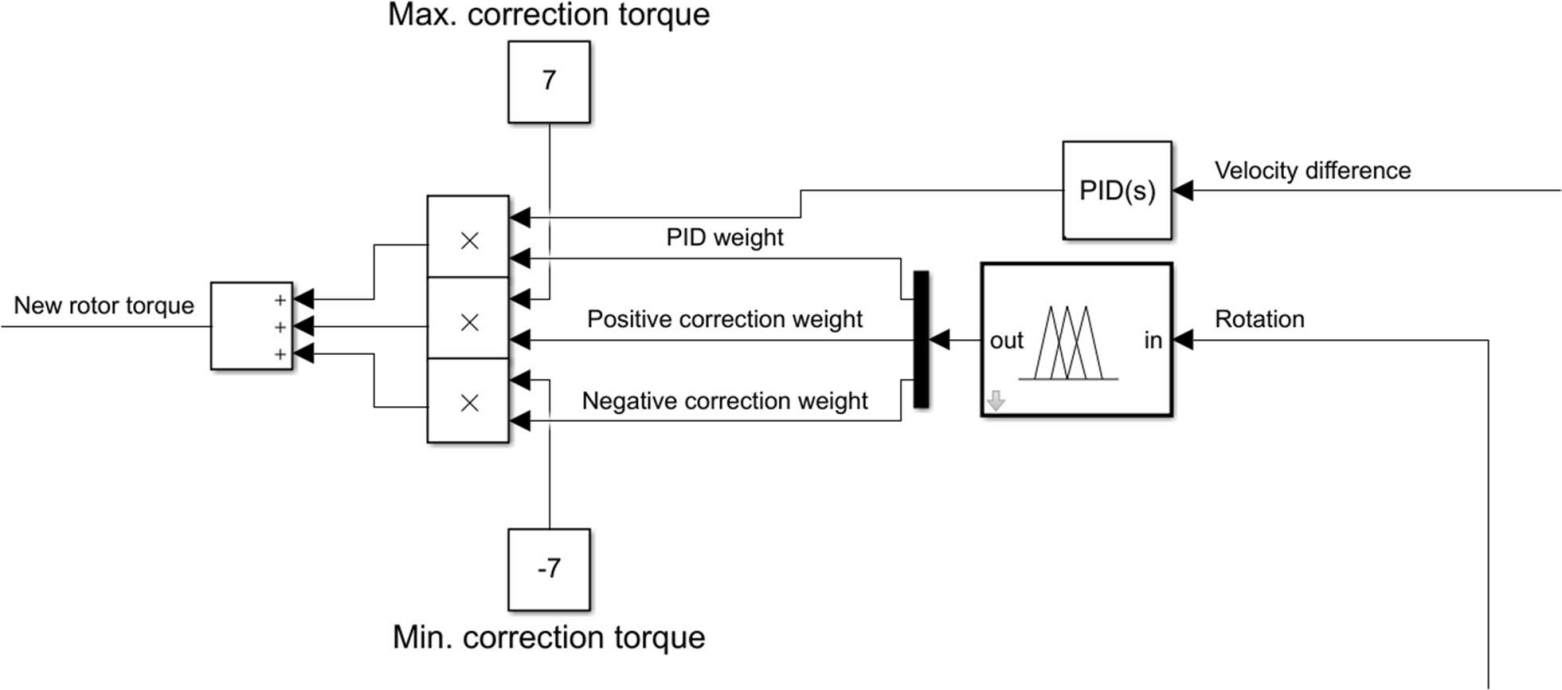
Fuzzy PID implementation in *Simulink*

The PID controller was set at the same parameters for every experimental case ($P=2$, $I=0.4$, $D=1$). Thanks to this, it is possible to analyse which of the cases introduces unacceptable behaviour of the system. The following fuzzy controller prevents the device from exceeding the patient’s range of motion. The algorithm analyses whether the position of the rotor enters the dangerous range, and if so, it corrects the control signal to act opposite to the end of the safe range. Its implementation in *Simulink* is presented in Fig. 5. Additionally, the maximum set torque of the motor was limited to $7\ Nm$ [37] while the maximum angular velocity of the rotor was assumed as $2.6\ \frac{rad}{s}$ [29].

Performance on the system is analysed by comparing time shapes of the signals of intended torques generated by the patient in the joint and the acceleration of the drive’s rotor. It is intended for the latter to be as proportional as possible to the former. Therefore, two following parameters describing the quality of the control method were defined:

- *Q* [$\left( m\cdot kg\right) ^{-1}$] - the ratio of the drive’s rotor and the intended torques generated by the patient;
- $t_{r_i}$ [*s*] - regulation times during which the value of *Q* parameter exceeds the range of $\left<\overline{Q}-\epsilon /2;\overline{Q}+\epsilon /2\right>$, where $\overline{Q}$ is the mean of the *Q* parameter for the session, and $\epsilon$ is the width of the acceptable ratio range, in this case, taken as $\epsilon =4\vert {\overline{Q}}\vert$.

**Fig. 6:**
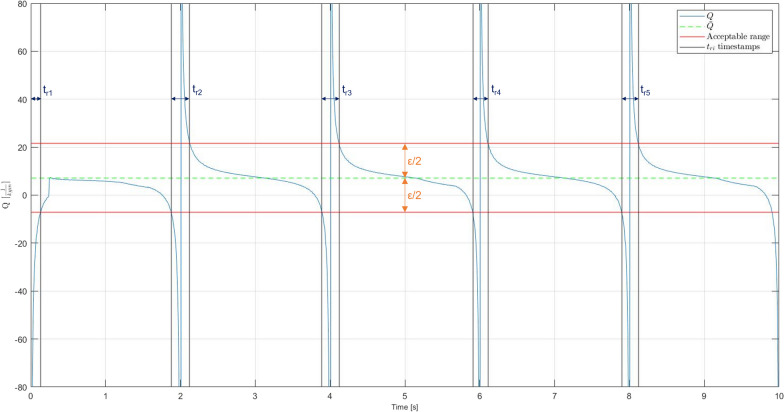
Quality parameters presented for one of the cases

An example of the time series for computed parameters is presented in Fig. 6. Intentionally, parameter *Q* should have the smallest deviation from $\overline{Q}$ in the acceptable ratio range, while the sum of the regulation times $\sum {t_{r_i}}^n_{i=1}$ and their maximum value $\max {\left( t_{r}\right) }$ should be minimised.

## Results and discussion

**Fig. 7:**
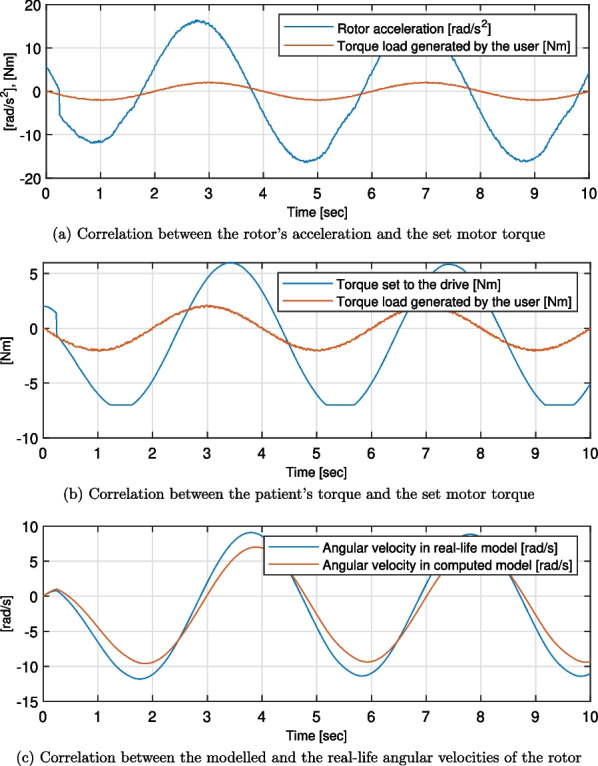
Results of the simulations for the test case 6, sinus patient’s torque waveform

**Fig. 8:**
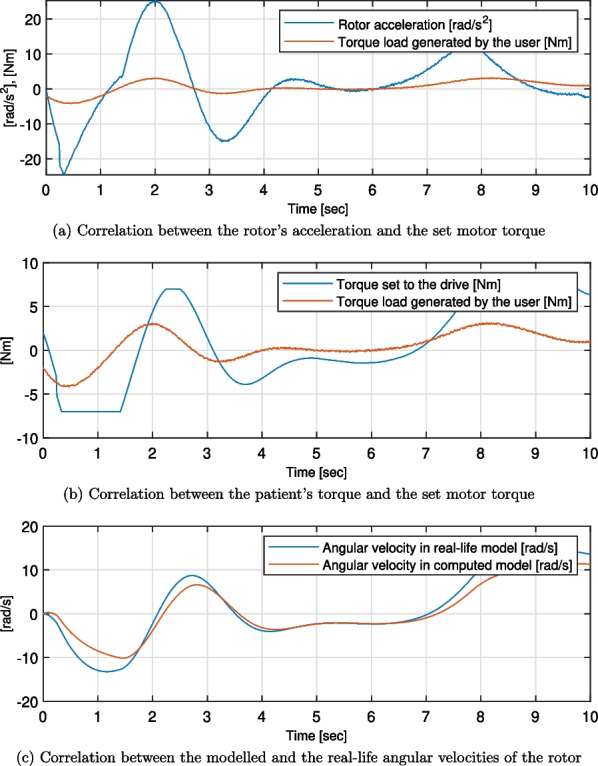
Results of the simulations for the test case 6, non-periodical randomised smooth patient’s torque waveform

**Fig. 9:**
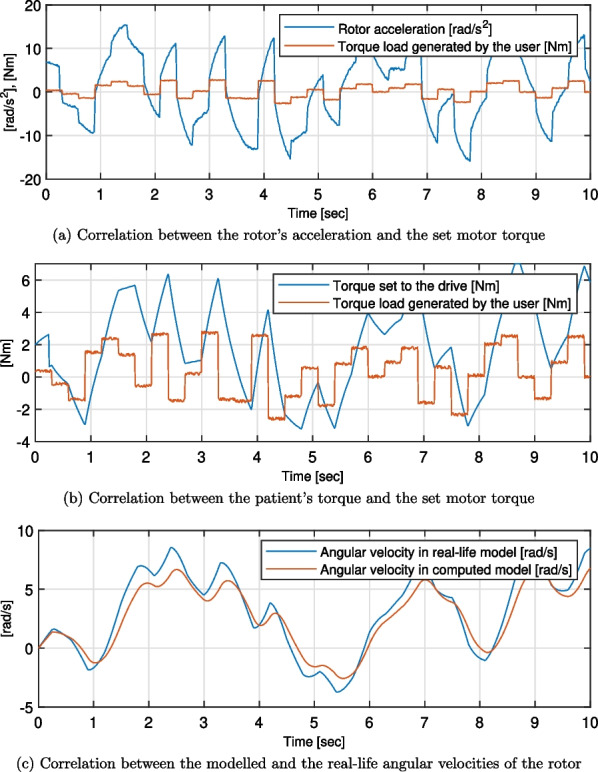
Results of the simulations for the test case 6, non-periodical randomised square patient’s torque waveform

Twenty-one simulation trials were conducted - for the combinations of seven test cases and three types of generated forms of patient torques. For all the simulation trials, torque set to the drive, rotor angular acceleration, and rotor angular velocities were computed. The first two were compared with the torque load generated by the user, while the last one was compared to the reference value of the idealised motor model. Exemplary plots of these are presented in Figs. 7, 8 and 9. Moreover, the quality metrics for all the trial was presented in Table 2. The average regulation times were not analysed as their values are always close to the average regulation times. This confirms that the regulation time after changing the direction of the torque exerted by the user is almost constant for every case.

**Table 2:** Quality metrics for all the test cases ($max\left( t_r\right) \ [s]$ - maximum value of the regulation times, $\sigma ^*(Q)\ [\left( m\cdot kg\right) ^{-1}]$ - average deviation from the $\overline{Q}$ in the acceptable range, not applicable placed if the *Q* parameter is out of the acceptable range for over 90% of the time)

Patients Torque	Sinus		Non-periodical randomised smooth		Non-periodical randomised square	
Test case	$Max\,\left( t_r\right)$	$\sigma ^*(Q)$	$Max\,\left( t_r\right)$	$\sigma ^*(Q)$	$Max\,\left( t_r\right)$	$\sigma ^*(Q)$
1	0.24	3.215	0.45	3.357	0.30	2.320
2	0.06	0.190	0.10	0.295	0.29	0.248
3	0.53	8.210	0.87	25.254	0.31	not applicable
4	0.92	1.637	0.52	3.218	0.31	2.781
5	0.08	2.289	0.15	2.800	0.31	1.925
6	0.28	3.216	0.59	3.858	0.33	2.189
7	0.28	3.218	0.81	3.761	0.37	2.264

During the tests, the angular velocity of rotors very occasionally exceeded its intended limits. However, it was accepted for a short-time period, as too large acceleration was never generated (assumed up to $19\ \frac{rad}{s^2}$).

The system’s behaviour tends to be similar for sinus and non-periodical randomised smooth waveforms of the user’s torque. For the square waveform, maximum regulation times remain close to 0.3 s, except for test case 7. The increased value of this indicator is the result of higher encoder inaccuracies.

Due to the modelling inertia moment of the reference system with the excessive value, the results of test case 3 were not acceptable. The set motor control torque was being limited to $\pm 7\ Nm$ almost within the whole timespan. However, bang-bang control is not applicable for exoskeletons, where immediate changes in dynamics can harm the disabled user.

Regardless of the case and the input patient’s torque waveform, the strong correlation between the shapes of the latter and both the set motor torque and the rotor angular acceleration was visible (see Figs. 7, 8 and 9). The regulation phases result in displacements of the corresponding graphs along the horizontal axis.

The main insights from the simulations are as follows:

- The differences between the intended torque and the one generated by the patient do not significantly affect the method’s performance. However, the noises of the encoder’s readings can strongly increase the regulation time.
- Precise modelling of the motor is not required. However, the differences between the real-life and the idealised model can increase deviations of *Q* parameters from its mean and the regulation times (compare cases 2-5 with 1, 6 and 7).
- The best results were obtained for undershoot inertia moments of the system and overshoot of its damping. Such cases had the shortest regulation times (even up to $0.06\ s$). Moreover, case 2 had the smallest deviation of *Q* parameters from its mean.
- The regulator’s parameters can directly affect the magnitude of the torque set to the motor. Therefore, they correlate with the level of supporting the patient. They should be adjusted to obtain the desired effect, e.g. to increase the velocities of the movements.

Test cases 2 and 5 resulted in the most favourable values of both quality indicators. Their combinations were used to continue the investigation to adjust the quasi-optimal PID parameters. As the input signals, only sinus and non-periodical randomised smooth waveforms of the user’s torque were used. This is due to the fact that rectangular waveforms were not differing the results significantly compared to smooth forms. An impact of *P* and *D* parameters on quality indicators and generated angular velocities was investigated. For all the simulations, a small difference between the real-life and the modelled damping ratio was assumed. The cases, however, differed in terms of the modelled moment of inertia. The results of trials are presented in Table 3.

**Table 3:** Test cases for experimental adjustment for PID settings ($J_m$ - value of the modelled *J* parameter, $J_r$ - value of the real-life *J* parameter, $B_m$ - value of the modelled *B* parameter, $B_r$ - value of the real-life *B* parameter, $max\left( t_r\right) \ [s]$ - maximum value of the regulation times, $\sigma ^*(Q)\ [\left( m\cdot kg\right) ^{-1}]$ - average deviation from the $\overline{Q}$ in the acceptable range, $max\left( \omega \right)$ - maximum measured angular velocity [rad/s])

No.	Input signal	*P*	*I*	*D*	$J_r$	$J_m$	$B_r$	$B_m$	$Max\,\left( t_r\right)$	$\sigma ^*\left( Q\right)$	$Max\,\left( \omega \right)$
1	Sinus	2	0.4	1	0.35	0.7	0.6	0.55	0.04	0.206	4.77
2	Sinus	10	0.4	1	0.35	0.7	0.6	0.55	0.06	0.384	6.84
3	Sinus	2	0.4	10	0.35	0.7	0.6	0.55	0.04	0.246	6.28
4	Non-periodical	2	0.4	1	0.35	0.7	0.6	0.55	0.11	0.431	7.08
5	Non-periodical	10	0.4	1	0.35	0.7	0.6	0.55	0.36	0.890	9.78
6	Non-periodical	2	0.4	10	0.35	0.7	0.6	0.55	0.18	0.690	9.22
7	Sinus	2	0.4	1	0.35	1.05	0.6	0.55	0.03	0.098	2.84
8	Sinus	10	0.4	1	0.35	1.05	0.6	0.55	0.03	0.120	3.50
9	Sinus	2	0.4	10	0.35	1.05	0.6	0.55	0.03	0.090	3.34
10	Non-periodical	2	0.4	1	0.35	1.05	0.6	0.55	0.06	0.171	4.18
11	Non-periodical	10	0.4	1	0.35	1.05	0.6	0.55	0.20	0.301	5.05
12	Non-periodical	2	0.4	10	0.35	1.05	0.6	0.55	0.12	0.258	4.88
13	Sinus	2	0.4	1	0.35	1.4	0.6	0.55	0.03	0.065	2.02
14	Sinus	10	0.4	1	0.35	1.4	0.6	0.55	0.02	0.062	2.36
15	Sinus	2	0.4	10	0.35	1.4	0.6	0.55	0.02	0.051	2.27
16	Non-periodical	2	0.4	1	0.35	1.4	0.6	0.55	0.03	0.089	2.97
17	Non-periodical	10	0.4	1	0.35	1.4	0.6	0.55	0.13	0.150	3.40
18	Non-periodical	2	0.4	10	0.35	1.4	0.6	0.55	0.07	0.137	3.31

Most of the obtained velocities exceed the set limit for the elbow joint of $2.6\ rad/s$ [29]. However, the test trials were used to assess the impact of the settings on obtained results.

Modification of the regulator settings has a direct impact on the torque set during the simulation. The data presented in Table 3 shows that increasing the *P* parameter increases the generated maximum speed of the system at the cost of increasing the average deviation of the *Q* parameter (observable while comparing the consecutive cases: 1 and 2, 4 and 5, 7 and 8, 10 and 11, 16 and 17). Raising the *D* parameter brings similar effects but the less visible. Moreover, the maximum regulation time is decreased for the periodic input signal. Thanks to this, the intention of a user is detected faster. As validated computationally, the parameters $P=2$, $I=0.4$ and $D=10$ give satisfactory results for all the tested cases.

**Fig. 10:**
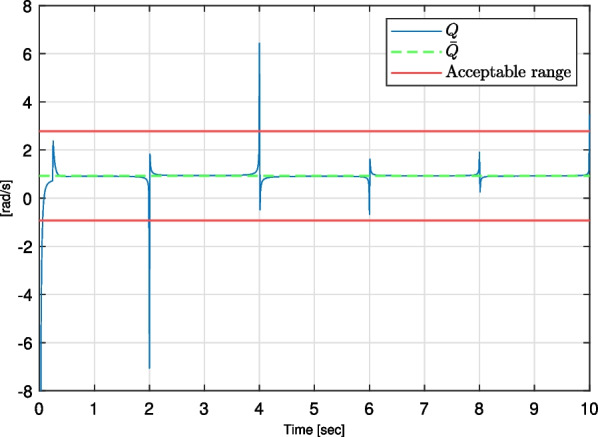
Quality parameters presented for the test case 15

Additionally, reducing the regulation time is possible by underestimating the system’s moment of inertia. Moreover, for such cases, the *Q* parameter time form is closer to constant (see Fig. 10). The settings with small *P* and *D* parameters were selected as quasi-optimal for the application, which does not require high velocities. On the contrary, if the system needs to move with higher dynamics, the *D* and, optionally, the *P* parameters should be increased.

Based on the data presented in Table 3, the developed method is robust and, hence, applicable to detecting patients’ intentions in exoskeleton-aided kinesiotherapy. As the method’s performance is presented for a single joint, it is applicable for any exoskeleton with the different number of joints. Its maximum regulation times remain in the range of 20-200 ms for 17 out of 18 cases (it is 360 ms only in case 5). These results are comparable to the reaction times obtained for EMG-based systems (130-300 ms) [38–41] and significantly better than the low-cost force sensor’s performance (410-1280 ms) [42, 43]. However, the results are worse in most cases than for the ultrasensitive pressure sensors (43 ms) [44].

Nevertheless, the proposed method does not increase the costs of the exoskeleton by adding EMG measuring systems or expensive sensors [38–41, 44]. Neither does it increase the mass and bulkiness of the structure as for the budget force sensors [42, 43].

According to the results, the method is applicable to use by patients with motor disorders. However, these can come with comorbidities such as spasticity or cognitive disorders.

In the first case, if not modified, the method would just enhance the muscle contraction and possibly harm the user. However, too big differences detected by the system could also be used for the emergency stop of the exoskeleton. Unfortunately, for all the testing settings, the reaction times $t_r$ would be significantly too long for the mentioned case.

On the contrary, not always there are contraindications to using the method for patients with cognitive disorders. Cases 1, 6 and 7 included potential differences between the intended motion and the motion realised with the musculoskeletal system ($M_l$ noise). However, as observed, the stronger differences can cause slower reactions of the system; for Parkinson’s disease, among others [45].

## Summary

The simulational investigation proved that the presented method is capable of detecting the user’s intentions without force sensors for exoskeleton-aided therapy. Two stages of the simulation were conducted. The first one universally assessed the method, while the second was used to analyse the impact of PID regulator parameters on the quality of control.

All of the obtained maximum regulation times remained below one second. However, the vast majority had a satisfactory value of 0.5 s or less, while the smallest recorded was 0.02 s. In the final set of simulations, the lowest average deviation of the *Q* parameter was 0.051.

The described method of controlling the drive in order to support the user’s movement intention is intended for implementation in the control system of the wearable device. Thanks to eliminating the force sensor from the design, the structure’s weight and manufacturing costs can be decreased. Hence, the system becomes easier to use for impaired patients and is available for society. The method implementation in real life is possible by creating an algorithm for the microcontroller or a microcomputer (in the ExoReha system - for the Raspberry Pi 4b). It is worth noting, however, that the created procedures must meet additional safety standards relating to the users’ dynamics. These were neglected during the presented simulations.

Optionally, the method can be enhanced by implementing the following modifications:

- extending the motor model by introducing its non-linearity and taking into account possible power transmission losses or measurement uncertainties;
- building the correlation model between the generated torque and the control current; however, the differences in the $K_t$ mechanical constant’s theoretical and real-life values can introduce errors to intention detection;
- combining the algorithm with the predictor based on the recurrent neural networks.

## Data Availability

The datasets used and/or analysed during the current study are available from the corresponding author on reasonable request.
